# Low density lipoprotein cholesterol and all-cause mortality rate: findings from a study on Japanese community-dwelling persons

**DOI:** 10.1186/s12944-021-01533-6

**Published:** 2021-09-12

**Authors:** Ryuichi Kawamoto, Asuka Kikuchi, Taichi Akase, Daisuke Ninomiya, Teru Kumagi

**Affiliations:** 1 Department of Community Medicine, Ehime University Graduate School of Medicine, Toon-city, Ehime 791-0295 Japan; 2 Department of Internal Medicine, Seiyo Municipal Nomura Hospital, 9-53 Nomura, Nomura-cho, Seiyo-city, Ehime 797-1212 Japan

**Keywords:** Low-density lipoprotein cholesterol, All-cause mortality, Community-dwelling persons, Cohort study

## Abstract

**Background:**

Low-density lipoprotein cholesterol (LDL-C) independently impacts aging-related health outcomes and plays a critical role in cardiovascular diseases (CVDs). However, there are limited predictive data on all-cause mortality, especially for the Japanese community population. In this study, it was examined whether LDL-C is related to survival prognosis based on 7 or 10 years of follow-up.

**Methods:**

Participants included 1610 men (63 ± 14 years old) and 2074 women (65 ± 12 years old) who participated in the Nomura cohort study conducted in 2002 (first cohort) and 2014 (second cohort) and who continued throughout the follow-up periods (follow-up rates: 94.8 and 98.0%). Adjusted relative risk estimates were obtained for all-cause mortality using a basic resident register. The data were analyzed by a Cox regression with the time variable defined as the length between the age at the time of recruitment and that at the end of the study (the age of death or censoring), and risk factors including gender, age, body mass index (BMI), presence of diabetes, lipid levels, renal function, serum uric acid levels, blood pressure, and history of smoking, drinking, and CVD.

**Results:**

Of the 3684 participants, 326 (8.8%) were confirmed to be deceased. Of these, 180 were men (11.2% of all men) and 146 were women (7.0% of all women). Lower LDL-C levels, gender (male), older age, BMI under 18.5 kg/m^2^, and the presence of diabetes were significant predictors for all-cause mortality. Compared with individuals with LDL-C levels of 144 mg/dL or higher, the multivariable-adjusted Hazard ratio (and 95% confidence interval) for all-cause mortality was 2.54 (1.58–4.07) for those with LDL-C levels below 70 mg/dL, 1.71 (1.15–2.54) for those with LDL-C levels between 70 mg/dL and 92 mg/dL, and 1.21 (0.87–1.68) for those with LDL-C levels between 93 mg/dL and 143 mg/dL. This association was particularly significant among participants who were male (*P* for interaction = 0.039) and had CKD (*P* for interaction = 0.015).

**Conclusions:**

There is an inverse relationship between LDL-C levels and the risk of all-cause mortality, and this association is statistically significant.

## Introduction

Numerous researchers have highlighted that low-density lipoprotein cholesterol (LDL-C) is a key risk factor associated with cardiovascular diseases (CVDs) [1]. Randomized controlled trials on the impact of lipid-lowering therapies have elucidated that reducing LDL-C levels lowers the risk of developing atherosclerotic CVD [2–5].

Research has offered contrasting findings on the association between LDL-C levels and CVD-related mortality. While some studies have shown a positive association [6], others present U-shaped associations [7]. Similarly, findings on the association between LDL-C levels and the risk of all-cause mortality remain contradictory. Whereas some research indicates a counterintuitive reverse association (increased levels of LDL-C reduce mortality) [8–11], others conclude that LDL-C levels are irrelevant [12, 13]. The varying results can be attributed to differences in the race, age, and gender of the targeted participants.

To address these inconsistencies, this study aimed to investigate whether LDL-C is related to survival prognosis based on 7 or 10 years of follow-up among Japanese community-dwelling persons.

## Methods

### Study design and participants

**Fig. 1:**
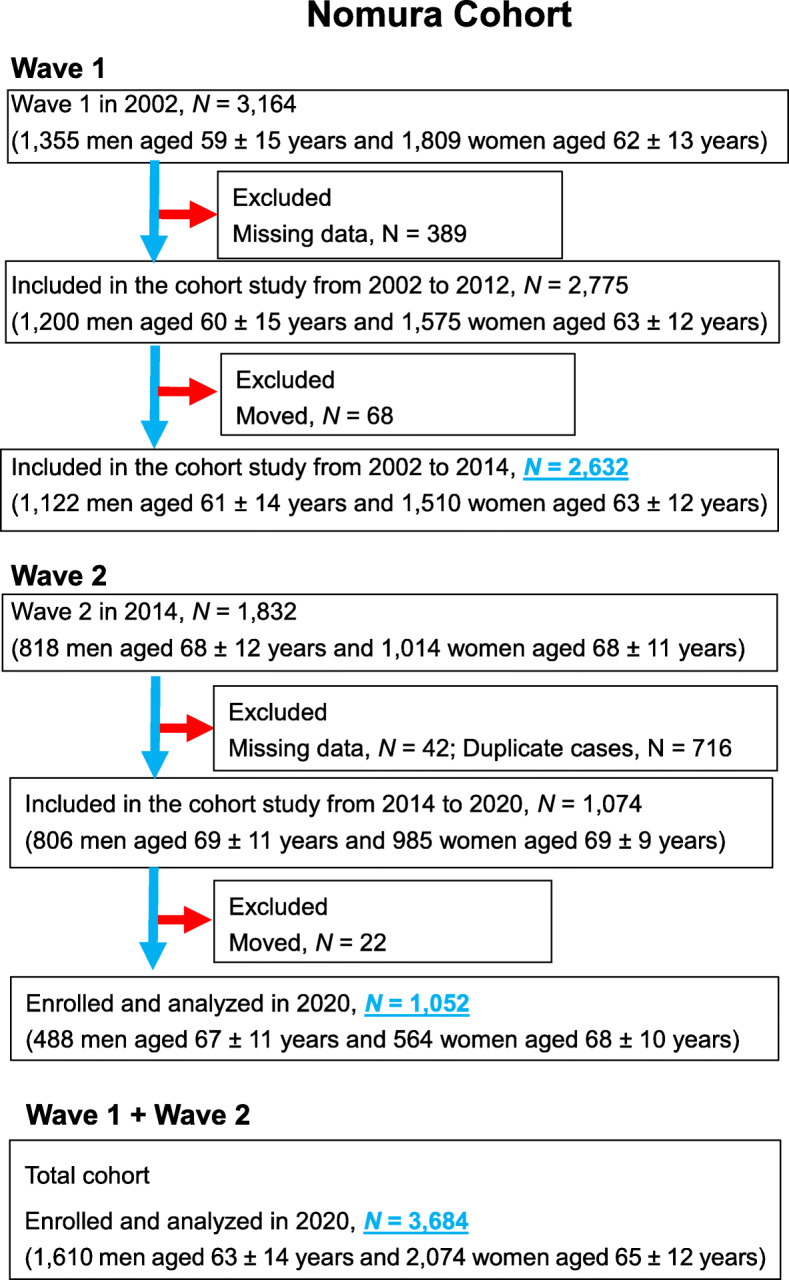
Flowchart of participants

This prospective cohort analysis is part of the Nomura studies [14] initiated in 2002 (first cohort) and 2014 (second cohort). Participants were recruited from the rural areas of Ehime Prefecture, Japan, with a focus on those who had undergone a community-based annual health check at the Nomura Health and Welfare Center. The first cohort included a total of 3164 people and the second cohort had 1832 people, all of whom were between the ages of 22 and 95 years. All procedures were carried out in accordance with relevant guidelines and regulations. A self-administered questionnaire was used to obtain data on participants’ physical activity, medical history, current condition, and medication. Figure 1 shows the flowchart for the inclusion and exclusion of participants. Follow-up assessments were conducted after a 10-year interval for the first cohort and a 7-year interval for the second cohort. Participants’ living status was confirmed using Japan’s Basic Resident Register. The Nomura studies did not provide information on causes of death or new onset of CVD [14]. This study examined evaluation data for the first and second cohorts (*N* = 3684). The study protocol was reviewed and approved by the Ehime University Hospital Institutional Review Board (IRB) (1903018). All participants provided written informed consent.

### Evaluation of risk factors

Demographic and risk factor data were obtained from clinical files. Body mass index (BMI) was estimated as weight (kg) divided by height (m^2^). Smoking status (packs-year) was calculated by multiplying the number of packs of cigarettes smoked per day by the number of years the person has smoked. Accordingly, participants were categorized as non-smokers, ex-smokers, light smokers (< 20 packs-year), or heavy smokers (≥ 20 packs-year). The Japanese liquor unit (22.9 g ethanol) was referenced to measure daily alcohol consumption. Participants were categorized as non-drinkers, occasional drinkers (< 1 unit/day), light daily drinkers (1–2 units/day), and heavy daily drinkers (2–3 units/day). None of the participants consumed more than 3 units/day. Systolic blood pressure (SBP) and diastolic blood pressure (DBP) were estimated by an automatic sphygmomanometer. To record both types of blood pressure, participants were asked to rest for at least 5 mins., after which an appropriately sized cuff was placed on the participant’s right upper arm while seated. The average of two consecutive measurements was used for the analysis. Triglyceride (TG), high-density lipoprotein cholesterol (HDL-C), LDL-C, serum uric acid (SUA), and blood glucose (BG) levels were measured during overnight fasting. The glomerular filtration ratio (eGFR) was estimated by modifying the chronic kidney disease epidemiology collaboration (CKD-EPI) equation with a Japanese coefficient: Male, Cr ≤ 0.9 mg/dl, 141 × (Cr/0.9) ^–0.411^ × 0.993 ^age^ × 0.813; Cr > 0.9 mg/dl, 141 × (Cr/0.9) ^–1.209^ × 0.993 ^age^ × 0.813; Female, Cr ≤ 0.7 mg/dl, 144 × (Cr/0.7) ^–0.329^ × 0.993 ^age^ × 0.813; Cr > 0.7 mg/dl, 144 × (Cr/0.7) ^–1.209^ × 0.993 ^age^ × 0.813 [15].

Participants were said to have hypertension if their SBP was 140 mmHg or higher, their DBP was 90 mmHg or higher, or they were on antihypertensive medication. Further, participants were classified as having hypertriglyceridemia if their TG levels were 150 mg/dL or higher and as having low HDL cholesterolemia if their HDL-C levels were below 40 mg/dL. Those on antidiabetic medication and with BG levels of 126 mg/dL or higher were categorized as diabetic. Use of a SUA-lowering medication or SUA levels of 7.0 mg/dL or higher were indicators of hyperuricemia. Chronic kidney disease (CKD) was defined as an eGFR under 60 mL/min/1.73 m^2^. Ischemic heart disease, ischemic stroke, and peripheral vascular disease were classified as CVD.

### Statistical analysis

All data were analyzed using IBM SPSS Statistics (version 26.0; SPSS Inc., Chicago, IL, United States). Normally distributed data are presented as the mean ± standard deviation and non-normally distributed data are expressed as median values (interquartile range) (e.g., for TG and BG levels). Parameters with non-normal distributions were log-transformed, and the transformed values were used in all analyses. Participants were divided into four groups on the basis of LDL-C level (very low: ≤ 69; low: 70–92; medium: 93–143; high: ≥ 144 mg/dL). Student’s *t*-test or an analysis of variance (ANOVA) was conducted on continuous data and a χ^2^ test was used on categorical data to analyze for differences in means and prevalence among the groups. Next, a Cox proportional hazard regression was performed to estimate hazard ratios (HRs) and 95% confidence intervals (CIs). The time variable was defined as the length between the age at the time of recruitment and that at the end of the study (the age of death or censoring). Analyses were adjusted for gender, age, BMI, smoking and drinking habits, history of CVD, hypertension, hypertriglyceridemia, low HDL-cholesterolemia, diabetes, CKD, use of lipid-lowering medication, and LDL-C group. Subgroup analyses were performed to determine if the observed association between LDL-C levels and all-cause mortality was consistent. Next, a likelihood ratio test was conducted to determine the interaction between LDL-C grouping and subgroup variables. All confounding variables, except the effect variable, were adjusted in the interaction test performed to analyze the effect variable. All *P*-values less than 0.05 were considered statistically significant.

## Results

The sample comprised 3684 participants. The mean age was 64 ± 13 years old and 43.7% were male. The median follow-up time (interquartile range) was 3160 (2330–3693) days. A total of 326 (8.8%) participants were confirmed to have died, and of these, 180 were men (11.2% of all men) and 146 were women (7.0% of all women) (Table 1).

**Table 1:** Baseline characteristics of participants

Characteristics ***N*** = 3684	Value
Gender (male), %	43.7
Age (years)	64 ± 13
Body mass index categories^a^, %	5.0/67.8/27.2
Body mass index (kg/m^2^)	23.3 ± 3.2
Smoking habits (non/ex/light/heavy), %	66.4/25.6/2.7/5.2
Drinking habits (non/occasional/light/heavy), %	51.7/25.8/13.6/9.0
History of cardiovascular disease, %	8.0
Hypertension, %	57.0
Systolic blood pressure (mmHg)	138 ± 21
Diastolic blood pressure (mmHg)	80 ± 11
Hypertriglyceridemia, %	18.0
Triglyceridemia (mg/dL)	93 (70–131)
Low HDL-cholesterolemia, %	4.4
HDL cholesterol (mg/dL)	63 ± 16
Lipid-lowering medication, %	9.7
LDL cholesterol (mg/dL)	118 ± 31
LDL cholesterol categories^b^, %	4.9/15.1/60.0/20.0
Diabetes, %	9.5
Blood glucose (mg/dL)	100 (91–114)
Chronic kidney disease, %	10.2
eGFR (mL/min/1.73 m^2^)	78.0 ± 16.4
Hyperuricemia, %	13.5
Serum uric acid (mg/dL)	5.1 ± 1.4

*HDL* high-density lipoprotein, *LDL* low-density lipoprotein, *eGFR* estimated glomerular filtration ratio

^a^ Body mass index categories: < 18.5 kg/m^2^, 18.5–25.0 kg/m^2^, ≥ 25 kg/m^2^

^b^ LDL cholesterol categories: very low, < 70 mg/dL; low, 70–92 mg/dL; medium, 93–143 mg/dL; high, ≥ 144 mg/dL

Data are presented as means ± standard deviation except for the data for triglycerides and hemoglobin A1c, which were skewed and are thus presented as median (interquartile range) values

Table 2 shows the baseline characteristics of the study participants according to LDL-C concentration at baseline. Age, SBP, and BG were higher, while eGFR and SUA levels were lower as LDL-C levels increased. The proportions of participants with hypertriglyceridemia, low HDL-cholesterolemia, and hyperuricemia were the highest in the very low LDL-C group (< 70 mg/dL). There were no statistically significant differences between LDL-C groups for other characteristics, including frequency of CVD, hypertension, diabetes, and CKD.

**Table 2:** Baseline characteristics of participants by low-density lipoprotein cholesterol categories

	LDL cholesterol categories (mg/dL)				
	Very Low (< 5%) < 70	Low (5–19%) 70–92	Medium (20–79%) 93–143	High (≥ 80%) ≥ 144	
Characteristics ***N*** = 3684	***N*** = 181	***N*** = 555	***N*** = 2212	***N*** = 736	***P***-value *
Gender (male), %	78.5	59.1	41.6	29.9	**< 0.001**
Age (years)	59 ± 17	61 ± 15	65 ± 12	65 ± 11	**< 0.001**
Body mass index categories ^a^, %	10.5/70.7/18.8	7.4/69.9/22.7	4.5/68.4/27.0	3.3/63.5/33.3	**< 0.001**
Body mass index (kg/m^2^)	22.3 ± 3.3	22.7 ± 3.2	23.3 ± 3.1	23.9 ± 3.3	**< 0.001**
Smoking habits (non = 1, ex = 2, light = 3, heavy = 4), %	43.6/50.3/1.7/4.4	55.1/35.3/2.5/7.0	68.8/22.7/3.0/5.5	73.6/20.9/2.2/3.3	**< 0.001**
Drinking habits (non = 1, occasional = 2, light = 3, heavy = 4), %	23.2/22.7/24.9/29.3	37.8/29.5/20.5/12.1	53.3/26.1/12.5/8.0	64.1/22.6/8.7/4.6	**< 0.001**
History of cardiovascular disease, %	6.1	8.6	8.5	6.7	0.303
Hypertension, %	53.6	52.3	58.1	58.3	0.057
Systolic blood pressure (mmHg)	136 ± 20	135 ± 21	138 ± 21	139 ± 22	**0.001**
Diastolic blood pressure (mmHg)	80 ± 12	79 ± 12	80 ± 11	81 ± 12	**0.001**
Hypertriglyceridemia, %	32.0	12.1	16.7	23.0	**< 0.001**
Triglyceridemia (mg/dL)	100 (60–179)	81 (61–114)	91 (69–127)	105 (81–145)	**< 0.001**
Low HDL-cholesterolemia, %	9.4	4.9	3.8	4.9	**0.004**
HDL cholesterol (mg/dL)	60 ± 19	63 ± 18	63 ± 15	61 ± 15	**< 0.001**
Lipid-lowering medication, %	6.6	11.0	10.5	6.9	**0.011**
LDL cholesterol (mg/dL)	58 ± 9	82 ± 6	118 ± 14	162 ± 18	**< 0.001**
Diabetes, %	11.0	8.5	9.7	9.4	0.733
Blood glucose (mg/dL)	97 (89–111)	98 (89–114)	100 (91–114)	103 (92–117)	**0.002**
Chronic kidney disease, %	11.6	9.0	10.2	10.6	0.712
eGFR (mL/min/1.73 m^2^)	82.9 ± 20.0	80.7 ± 17.4	77.6 ± 16.1	76.2 ± 15.1	**< 0.001**
Hyperuricemia, %	26.5	15.0	12.5	12.2	**< 0.001**
Serum uric acid (mg/dL)	5.8 ± 1.6	5.2 ± 1.5	5.1 ± 1.4	5.1 ± 1.4	**< 0.001**

^a^ Body mass index categories: < 18.5 kg/m^2^, 18.5–25.0 kg/m^2^, ≥ 25 kg/m^2^. Data presented are means ± standard deviation. Data for triglycerides and Hemoglobin A1c were skewed and are thus presented as median (interquartile range) values, and were log-transformed for analysis. *P-values are from ANOVA for continuous variables or from the χ^2^-test for categorical variables. Significant values (*P* <  0.05) are presented in bold

**Fig. 2:**
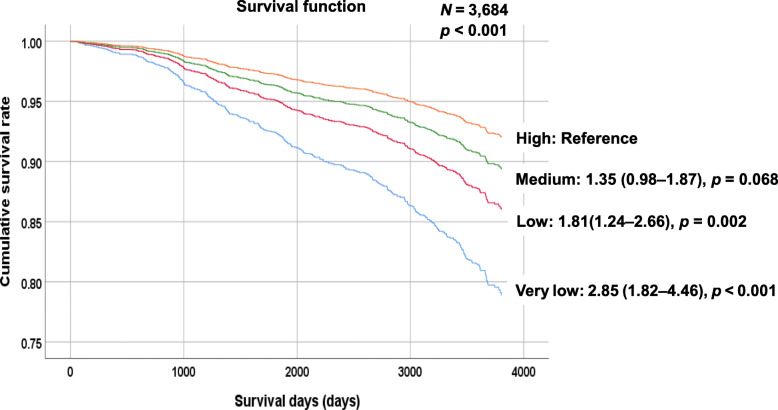
Analysis of the association between low-density lipoprotein cholesterol groups and all-cause mortality during the follow-up period using a survival function. *P*-values were obtained through a log-rank test of equality across various strata

Kaplan–Meier survival curves were charted for survival days and cumulative survival rates to identify patterns in the relationships between the four LDL-C groups and all-cause mortality (Fig. 2). The cumulative survival rate was significantly lower for individuals with LDL-C levels that were very low (< 70 mg/dL) or low (93–143 mg/dL) compared with those with high LDL-C levels (≥ 144 mg/dL) (HR: *P* = 0.002 and *P* <  0.001, respectively).

The HRs and 95% CIs for the quantitative and categorical variables that were identified as predictors of mortality in the multivariable analysis are presented in Table 3. Among the variables in the model, significant predictors for all-cause mortality were gender (male), older age, BMI under 18.5 kg/m^2^, lower LDL-C levels, and diabetes***.*** Compared with individuals with high LDL-C levels (≥ 144 mg/dL), the multivariable-adjusted HR (95% CI) for all-cause mortality was 2.54 (1.58–4.07) for those with very low LDL-C levels (< 70 mg/dL), 1.71 (1.15–2.54) for those with low LDL-C levels (70–92 mg/dL), and 1.21 (0.87–1.68) for those with medium LDL-C levels (93–143 mg/dL).

**Table 3:** Multivariable-adjusted hazard ratios and 95% confidence intervals of baseline characteristics for all-cause mortality

Characteristics ***N*** = 3684		HR (95% CI)	***P***-value
Gender (female = 1, male = 2)	2 vs 1	1.43 (1.04–1.98)	**0.027**
Age (per 1 year)	–	1.09 (1.08–1.11)	**< 0.001**
Body mass index categories (< 18.5 kg/m^2^ = 1, 18.5–24.9 kg/m^2^ = 2, ≥ 25.0 kg/m^2^ = 3)	2 vs 1 3 vs 1	0.51 (0.35–0.75) 0.40 (0.26–0.63)	**0.001** **< 0.001**
Smoking habits (non = 1, ex = 2, light = 3, heavy = 4)	2 vs 1 3 vs 1 4 vs 1	1.29 (0.97–1.72) 0.34 (0.11–1.07) 0.97 (0.50–1.90)	0.085 0.064 0.934
Drinking habits (non = 1, occasional = 2, light = 3, heavy = 4)	2 vs 1 3 vs 1 4 vs 1	1.02 (0.76–1.38) 0.85 (0.59–1.23) 0.95 (0.61–1.49)	0.880 0.394 0.834
History of cardiovascular disease (no = 1, yes = 2)	2 vs 1	1.33 (0.99–1.80)	0.061
Hypertension (no = 1, yes = 2)	2 vs 1	1.09 (0.84–1.40)	0.528
Hypertriglyceridemia (no = 1, yes = 2)	2 vs 1	0.92 (0.67–1.26)	0.604
Low HDL-cholesterolemia (no = 1, yes = 2)	2 vs 1	0.54 (0.29–1.00)	0.052
LDL cholesterol categories (< 70 mg/dL = 1, 70–92 mg/dL = 2, 93–143 mg/dL = 3, ≥ 144 mg/dL = 4)	1 vs 4 2 vs 4 3 vs 4	2.54 (1.58–4.07) 1.71 (1.15–2.54) 1.21 (0.87–1.68)	**< 0.001** **0.008** 0.250
Lipid-lowering medication (no = 1, yes = 2)	2 vs 1	0.89 (0.60–1.31)	0.551
Diabetes (no = 1, yes = 2)	2 vs 1	1.66 (1.23–2.23)	**0.001**
Chronic kidney disease (no = 1, yes = 2)	2 vs 1	0.93 (0.69–1.27)	0.659
Hyperuricemia (no = 1, yes = 2)	2 vs 1	1.17 (0.85–1.60)	0.337

*HR* hazard ratio, *CI* confidence interval, *vs* versus. Significant values (*P* <  0.05) are presented in bold

Table 4 shows a higher prevalence of all-cause mortality in the very low and low LDL-C groups compared with the high LDL-C group. Next, participants were divided into four groups by age (< 55, 55–64, 65–74, and ≥ 75 years), and the analysis was adjusted for gender, age, BMI, smoking and drinking status, history of CVD, hypertension, hypertriglyceridemia, low HDL-cholesterolemia, use of lipid-lowering medication, CKD, and hyperuricemia. In participants below 55 years of age, there were no significant associations between the four LDL-C groups. In participants aged 55 years and older, very low LDL-C levels (< 70 mg/dL) were significantly associated with an increased risk of all-cause mortality, and among those aged 75 years and older, even those with LDL-C below 92 mg/dl had an increased risk.

**Table 4:** Hazard ratios and 95% confidence intervals of baseline low density lipoprotein cholesterol categories for all-cause mortality by age group

LDL cholesterol	Prevalence of death/total (%)	Non-adjusted HR (95% CI)	Gender and age-adjusted HR (95% CI)	Multivariable-adjusted HR (95% CI) ^**a**^
**< 55 years of age (*n*** **= 788)**				
Very low	4/65 (6.2)	7.03 (0.79–62.9)	6.47 (0.70–59.8)	4.91 (0.49–49.6)
Low	1/168 (0.6)	0.71 (0.04–11.3)	0.70 (0.04–11.3)	0.65 (0.04–10.9)
Medium	13/431 (3.0)	3.65 (0.48–27.9)	4.03 (0.52–30.9)	3.19 (0.40–25.4)
High	1/124 (0.8)	1.00	1.00	1.00
*P*-value	**0.041**	0.108	0.123	0.226
**≥ 55 and < 65 years of age (*n*** **= 783)**				
Very low	4/28 (14.3)	**8.19 (1.83–36.6)**	**6.66 (1.40–31.7)**	**7.04 (1.40–35.5)**
Low	9/99 (9.1)	**5.74 (1.55–21.2)**	**4.66 (1.22–17.8)**	**4.33 (1.12–16.8)**
Medium	19/468 (4.1)	2.55 (0.75–8.62)	2.29 (0.67–7.80)	2.24 (0.65–7.65)
High	3/188 (1.6)	1.00	1.00	1.00
*P*-value	**0.002**	**0.009**	**0.042**	0.057
**≥ 65 and < 75 years of age (*n*** **= 1395)**				
Very low	9/52 (17.3)	**2.76 (1.24**–**6.15)**	**2.34 (1.02**–**5.39)**	**2.52 (1.08**–**5.93)**
Low	18/179 (10.1)	1.62 (0.84–3.11)	1.43 (0.73–2.79)	1.37 (0.70–2.69)
Medium	60/872 (6.9)	1.12 (0.66–1.89)	1.02 (0.60–1.74)	0.93 (0.54–1.59)
High	18/292 (6.2)	1.00	1.00	1.00
*P*-value	**0.007**	**0.037**	0.109	**0.044**
**≥ 75 years of age (*n*** **= 718)**				
Very low	16/36 (44.4)	**3.03 (1.61**–**5.71)**	**2.53 (1.33**–**4.84)**	**2.13 (1.09**–**4.16)**
Low	34/109 (31.2)	**2.27 (1.35**–**3.84)**	**1.99 (1.17**–**3.39)**	**1.75 (1.01**–**3.05)**
Medium	93/441 (21.1)	1.27 (0.81–1.98)	1.22 (0.78–1.92)	1.09 (0.68–1.73)
High	24/132 (18.2)	1.00	1.00	1.00
*P*-value	**< 0.001**	**< 0.001**	**0.004**	**0.021**

*HR* hazard ratio, *CI* confidence interval, *LDL* cholesterol categories: very low, < 70 mg/dL; low, 70–92 mg/dL; medium, 93–143 mg/dL; high, ≥ 144 mg/dL

^a^ Multivariate-adjusted HR: adjusted for gender, age, body mass index categories, smoking status, drinking status, history of cardiovascular disease, hypertension, hypertriglyceridemia, low HDL-cholesterolemia, lipid-lowering medication, chronic kidney disease, and hyperuricemia

Significant values (*P* <  0.05) are presented in bold

Lastly, participants were stratified in Table 5 by gender, BMI (< 25 and ≥ 25 kg/m^2^), history of CVD, hypertension, diabetes, and CKD status, use of lipid-lowering medication, and time to death (< 1095 or ≥ 1095 days). Overall, the results showed that very low (< 70 mg/dL) and low (70–92 mg/dL) LDL-C levels were associated with a higher risk of all-cause mortality, and this association was particularly significant among participants who were male (*P* = 0.039 for interaction) and had CKD (*P* = 0.015 for interaction).

**Table 5:** Hazard ratios and 95% confidence intervals of baseline low density lipoprotein cholesterol categories for all-cause mortality by sub-analysis

Characteristics ***N*** = 3684	Multivariable-adjusted HR (95% CI)			***P***-value	***P*** for interaction
	Very low	Low	Medium		
Gender					
Male (*n* = 1610)	**2.80 (1.53–5.14)**	**1.84 (1.05–3.22)**	0.98 (0.58–1.66)	**< 0.001**	**0.039**
Female (*n* = 2074)	1.42 (0.42–4.74)	1.41 (0.73–2.70)	1.44 (0.94–2.19)	0.407	
Body mass index					
< 25 kg/m^2^ (*n* = 2681)	**2.43 (1.46–4.05)**	**1.66 (1.07–2.58)**	1.02 (0.70–1.48)	**< 0.001**	0.261
≥ 25 kg/m^2^ (*n* = 1003)	2.36 (0.62–9.04)	1.46 (0.54–3.94)	1.89 (0.94–3.79)	0.308	
History of cardiovascular disease					
No (*n* = 3389)	**2.63 (1.59–4.35)**	**1.62 (1.04–2.50)**	1.15 (0.81–1.64)	**< 0.001**	0.907
Yes (*n* = 295)	1.64 (0.39–6.93)	**3.03 (1.03–8.84)**	1.41 (0.52–3.82)	0.099	
Hypertension					
No (*n* = 1583)	**3.19 (1.35–7.56)**	1.71 (0.80–3.66)	1.32 (0.71–2.45)	**0.044**	0.705
Yes (*n* = 2101)	**2.35 (1.33–4.16)**	**1.80 (1.13–2.88)**	1.16 (0.79–1.70)	**0.002**	
Lipid-lowering medication					
No (*n* = 3327)	**2.75 (1.66–4.55)**	**2.06 (1.35–3.14)**	1.31 (0.92–1.86)	**< 0.001**	0.379
Yes (*n* = 357)	3.74 (0.91–15.4)	0.31 (0.08–1.17)	0.49 (0.19–1.28)	**0.003**	
Diabetes					
No (*n* = 3334)	**3.32 (2.00–5.52)**	**1.85 (1.19–2.86)**	1.21 (0.84–1.75)	**< 0.001**	0.059
Yes (*n* = 350)	0.78 (0.19–3.17)	1.21 (0.45–3.24)	1.04 (0.48–2.27)	0.937	
Chronic kidney disease					
No (*n* = 3310)	**1.92 (1.11–3.32)**	1.48 (0.96–2.28)	1.04 (0.73–1.47)	**0.017**	**0.015**
Yes (*n* = 374)	**8.69 (2.77–27.3)**	**4.89 (1.62–14.7)**	2.50 (0.94–6.59)	**< 0.001**	
Time to death					
< 1095 days (n = 73)	Not examined	Not examined	Not examined	–	Not examined
≥ 1095 days (*n* = 3611)	**2.68 (1.67–4.28)**	**1.74 (1.17–2.59)**	1.20 (0.86–1.66)	**< 0.001**	

*HR* hazard ratio, *CI* confidence interval, *LDL* cholesterol categories: very low, < 70 mg/dL; low, 70–92 mg/dL; medium, 93–143 mg/dL; high, ≥ 144 mg/dL = Reference

^a^ Multivariate-adjusted HR: adjusted for gender, age, body mass index categories, smoking status, drinking status, history of cardiovascular disease, hypertension, hypertriglyceridemia, low HDL-cholesterolemia, lipid-lowering medication, chronic kidney disease, and hyperuricemia. Significant values (*p* < 0.05) are presented in bold

## Discussion

The main finding of this cohort study is that LDL-C is a significant and independent predictor of all-cause mortality in community-dwelling adults. After adjustment for possible confounding factors, the results showed that participants with the very low LDL-C levels (< 70 mg/dL) were at a significantly higher risk for all-cause mortality than those with high LDL-C levels (≥ 144 mg/dL). To the best of our knowledge, few studies have demonstrated the relationship between LDL-C level and all-cause mortality in Japanese community-dwelling persons.

The results of this study, especially that low LDL-C levels are significantly associated with an increased risk of all-cause mortality, are consistent with the results of several existing studies. The Kangbuk Samsung Health Study on 347,971 individuals (mean age: 39.6 years old; male: 57.4%; mean follow-up: 5.64 ± 3.27 years) highlighted that the lowest LDL-C group (< 70 mg/dL) was at a higher risk of all-cause mortality (HR: 1.81; 95% CI: 1.44–2.28) compared with the reference group (120–139 mg/dL) [7]. Further, Johannesen et al. [16] reported 2028 deaths among the total number of participants and 11,376 (10.5%) deaths during the study among the 108,243 individuals aged 20–100 years (male: 45.0%; median follow-up: 9.4 years). The study also showed a U-shaped relationship between LDL-C levels and the risk of all-cause mortality: that is, low levels (< 70 mg/dL; HR: 1.25, 95% CI: 1.15–1.36) and high levels (> 189 mg/dL; HR: 1.15, 95% CI: 1.05–1.27) were associated with an increased risk of all-cause mortality compared with the reference group (132–154 mg/L). The China Health and Retirement Longitudinal Study (follow-up: 4 years) recorded a total of 305 deaths out of 4981 male participants. Compared with the LDL-C baseline group (117–137 mg/dL), a lower LDL-C level (≤ 84 mg/dL) was associated with an increased risk of four-year all-cause mortality in middle-aged and older adult Chinese male participants [17]. According to a recent systematic review of 19 cohort studies with more than 68,094 older adults, all-cause mortality was highest in the lowest LDL-C quartile group [18]. In addition, a population-based register study on 118,160 individuals aged 50 years or older without baseline statin use showed an association between high LDL-C levels and lower mortality among older adults [10]. The present study also reports that low LDL-C levels at baseline, as well as being male, older, having lower BMI, and having a history of diabetes, were linked with an increase in all-cause mortality. The association between LDL-C levels and all-cause mortality was particularly significant for male participants and those who have CKD. The opposite trend was not observed in people with a BMI of 25 kg/m^2^ or higher, those with a history of CVD, or those with diabetes. Higher LDL-C levels are often observed in these patient groups [19, 20], but this does not necessarily lead to increased mortality [21]. In addition, for those with low LDL-C levels due to lipid-lowering medication, all-cause mortality was not significantly increased; for these individuals, low LDL-C levels were intentional, rather than an indicator of poor prognosis. However, in the study with 118,160 participants, it was reported that those with the highest LDL-C levels lived longer than those on lipid-lowering medication [10].

The study examined the association between LDL-C levels and mortality outcomes in a real-world setting among community-dwelling individuals, including those on lipid-lowering therapy and those with a baseline history of CVD, hypertension, or diabetes. However, the sub-analysis shows similar notable findings for those who were not undergoing lipid-lowering therapy or had no other diseases. The findings suggest that considerably lower LDL-C levels do not necessarily protect against all-cause mortality among community-dwelling persons who are not on lipid-lowering medication, thus supporting the lipid paradox [7]. In addition, the difference in results between male and female participants can be attributed to fewer deaths among women than men, which results in insufficient association power [17]. We believed that baseline serum LDL-C levels would be positively associated with CVD [22] and mortality in middle-aged people. On the other hand, a negative association between LDL-C levels and all-cause mortality has been observed in the elderly [23, 24]. In this population, mortality from non-CVD increased with decreasing LDL-C, which could be due to malnutrition or infectious diseases [25–28]. This would explain the reversed association between LDL-C levels and all-cause mortality that was only observed in the older population. We discuss this further in the following paragraph. In our study, the reversed association was consistently found in the age group of 55 years and older, but no significant association was found among the four LDL-C groups in participants younger than 55 years.

The mechanisms leading to increased all-cause mortality in individuals with very low LDL-C levels are not completely understood. Several explanations can be offered for these findings. Low LDL-C levels increase susceptibility to serious diseases [29]. Conversely, it has been hypothesized that frailty and illnesses lower cholesterol levels [30]. The latter concerns that cholesterol may be lowered by serious diseases shortly before death could be dispelled because mortality during the 3 years of observation were excluded in the current study. LDL-C may protect against viruses and cancers caused by viruses, and is therefore a component of innate immunity [26]. Ravnskov et al. [27] reviewed nine cohort studies involving more than 140,000 individuals. The review showed that the occurrence of cancer was inversely associated with cholesterol levels measured 10–30 years earlier, and the association remained when cancer cases that appeared during the first 4 years were excluded. Moreover, the lowering of lipids in rodents have led to cancer in previous experiments [25]. In addition, exotoxins produced by Gram-positive bacteria are absorbed by LDL-C [31]. Thus, higher LDL-C levels have been associated with reduced infection-related mortality and other non-CVD mortality, which explains the inverse relationship with all-cause mortality [28]. In this study, comorbidities such as hypertriglyceridemia, low HDL cholesterolemia, and hyperuricemia were more frequently observed in individuals with the lowest LDL-C levels. Finally, the relationship between LDL-C and mortality from coronary artery disease has also been reported in a paradoxical hypothesis regarding the relationship with mortality, and patients with higher LDL-C at admission had not higher all-cause mortality compared to patients with normal or low LDL-C [32, 33]. If high LDL-C were the cause, the effect should have been the opposite.

### Study strengths and limitations

The strengths of this study are the fact that it is a long-term follow-up collection, the sample size, the adjustment for possible confounding factors, and the inclusion of sensitivity analyses. However, the authors acknowledge some limitations. First, the sample consisted mainly of relatively healthy middle-aged and elderly people (mean age: 64 ± 13 years) who lived in rural areas of Japan and participated in the health checkup. Therefore, it cannot be considered representative of the general population. Second, the survey covered people whose deaths were registered in the basic resident register. Those who moved out of the region during the survey period are not included. Third, the possible effects of medication (e.g., antihypertensive, lipid-lowering, and antidiabetic medication), underlying diseases, and lifestyle modifications at the baseline and during the follow-up period on the present findings cannot be overlooked. Fourth, the threshold for the high LDL-C group may have been too low to evaluate the U-shaped relationship between LDL-C levels and all-cause mortality. If high LDL-C was defined as 190 mg/dL or higher in this study, the high LDL-C group would have included 52 individuals, with a 7.7% mortality rate during the observation period, which was not significantly different from the reference value (data not shown). Fifth, this study did not measure certain specific lipoproteins (e.g., small dense LDL), which could be a possible explanation for this phenomenon. Finally, the relatively low number of participants and deaths may weaken the causal relationship between LDL-C levels and all-cause mortality.

## Conclusions

The current results, based on a follow-up study of people aged 22 years and older, show that having very low LDL-C levels (< 70 mg/dL) is predictive of higher all-cause mortality, after adjustment for potential confounders such as body composition indices and metabolic factors. Therefore, further attention to individuals for whom lower LDL-C levels are not induced by lipid-lowering medication may be necessary.

## Data Availability

The participant survey data supporting the conclusions of this manuscript are not publicly available due to protection of the privacy of the participants.
